# Advancement in the Management of Synchronous Colorectal Liver Metastasis: A Comprehensive Review of Surgical, Systemic, and Local Treatment Modalities

**DOI:** 10.7759/cureus.80860

**Published:** 2025-03-19

**Authors:** Syed Hassan Raza Bokhari, Muhammad Hammad Muzaffar, Basil Ahmad, Soondas Waheed, Shabab U Rehman, Komal Syed

**Affiliations:** 1 Trauma and Orthopedics, Dudley Group of Hospitals, Dudley, GBR; 2 Surgery, Kimshealth Medical Center, Riyadh, SAU; 3 Intensive Care Unit, Hameed Latif Hospital, Lahore, PAK; 4 Emergency Medicine, Mid City Hospital, Lahore, PAK; 5 General Surgery, Hayatabad Medical Complex, Peshawar, PAK; 6 Pharmacy, Hamdard University, Lahore, PAK

**Keywords:** minimally invasive surgery, neoadjuvant chemotherapy, personalized treatment strategies, staged resection, synchronous colorectal liver metastases (scrlm)

## Abstract

Synchronous colorectal liver metastases (sCRLMs) present a complex therapeutic challenge requiring multimodal management strategies due to their complex tumor biology, variable disease progression, and the need to balance oncologic control with liver function preservation. This systematic review evaluates recent advancements in surgical approaches, systemic therapies, and local treatment modalities. A comprehensive literature search was conducted across major databases (PubMed, EMBASE, and Cochrane) for studies published between 2013 and 2024. Studies evaluating surgical techniques, perioperative outcomes, systemic therapy integration, and local treatments for sCRLM were included. Quality assessment was performed using the Newcastle-Ottawa Scale for observational studies and the Cochrane risk-of-bias tool for randomized controlled trials (RCTs). Analysis of nine primary studies encompassing 3,856 patients revealed evolving treatment paradigms. This review includes English-language studies (2013-2024) on adult patients (≥18 years) with sCRLM, covering RCTs, cohort, and case-control studies reporting survival, perioperative outcomes, or quality of life (≥12 months follow-up). Exclusions include case reports (<10 patients), metachronous metastases, conference abstracts, reviews without data, unclear methodology, duplicates, and animal/in-vitro studies. Staged resection demonstrated superiority over the simultaneous approach in recent analyses (win ratio 1.59, 95%CI 1.47-1.71). This indicates that patients undergoing staged resection had a 59% higher likelihood of achieving better outcomes (such as survival or fewer complications) compared to those undergoing simultaneous resection. The narrow confidence interval suggests strong statistical reliability of this finding. Minimally invasive techniques showed comparable outcomes to open surgery, with acceptable morbidity rates (21.7%) even in simultaneous resections. Neoadjuvant chemotherapy with targeted agents achieved high resectability rates (97%) and significant response rates (66%). The presence of liver metastases negatively impacted immunotherapy efficacy, suggesting the need for tailored approaches. Management of sCRLM has evolved toward a more personalized approach incorporating advanced surgical techniques, targeted therapies, and novel treatment sequencing. While staged resection may offer advantages in selected cases, treatment decisions should be individualized based on patient and disease characteristics. Future research should focus on optimizing patient selection and treatment sequencing through prospective trials.

## Introduction and background

The global burden of colorectal cancer (CRC) represents a significant public health challenge, with liver metastases being the leading cause of death in this patient [1,2]. Approximately 25% of patients present with synchronous colorectal liver metastases (sCRLMs) at initial diagnosis, while an additional 25%-35% develop metachronous liver metastases during their disease course [3]. The presence of synchronous metastases has traditionally been associated with poor prognosis, with historical five-year survival rates below 5% when treated with palliative intent alone [4]. However, the landscape of sCRLM management has undergone revolutionary changes over the past two decades, driven by advances in surgical techniques, systemic therapies, and local treatment modalities [5].

sCRLM is when cancer from the colon or rectum spreads to the liver. Often, CRC's first place to spread is the liver, and when it does spread there, it can result in the formation of multiple growths all over the liver growths that impair the liver's ability to function as it should. The growths vary in size, with some being so large or so many that they cannot be surgically removed; it poses a huge problem in cancer treatment, necessitating a team of many specialists to work together [6]. Surgical intervention is the most efficient means of getting rid of sCRLM but performing surgery is not often a viable option. Because the sCRLM is mainly so well established in the liver, the liver is not considered functionally alive without the tumors [7]. Liver resection would also condemn the patient to death. Thus, a combination of systemic therapies and conversion therapies of sCRLM combined with surgical interventions is necessary [8].

In sCRLM, conversion therapy, volumetric analysis, and multidisciplinary oncologic management are key to effective treatment [9]. Conversion therapy aims to shrink or stabilize initially unresectable tumors, making them eligible for surgical removal. This might include chemotherapy or radiation therapy [10]. Volumetric analysis utilizes imaging techniques to determine how big the tumors are and how much of the liver is involved. If the tumor burden is too large, then conversion therapy is next on the agenda [11]. Multidisciplinary oncologic management, with a team of specialists (e.g., hepatobiliary surgeons, oncologists, and radiologists) making the call on what happens next [12].

sCRLM occurs when CRC spreads to the liver via the portal venous system, often during the tumor's early progression. The liver's unique blood supply makes it highly prone to being a metastasis target. Its dual blood supply (from both the portal vein and the hepatic artery) makes the liver a highly vascularized organ, a poor interstitial environment for tumor cells trying to establish a foothold [13]. Liver tumors often succeed by rapidly recruiting blood vessels (angiogenesis) as they grow and by very effectively and sometimes replacing the normal liver architecture with tumor cells [14].

The simultaneous and staged resection for sCRLMs is the preferred treatment method; the choice between these two forms of treatment depends on multiple factors, including tumor burden, patient comorbidities, and surgical risk [15]. Simultaneous resection is generally preferred in patients with limited liver disease, good functional status, and a low expected risk of postoperative liver failure. This is where both the primary colorectal tumor and liver metastases are removed in one procedure. This method shortens the entire course of treatment, cutting down on the time the patient has to spend in a hospital and lessening the overall effect of the disease on the patient’s life. It does, however, carry a higher risk of complications around the time of an operation, especially in people who are having large parts of the liver removed [16].

Patients with a high tumor burden, serious comorbidities, or an insufficient future liver remnant may be better served by staged resection strategies such as the classical, reverse, or liver-first approaches. These strategies allow systemic therapy to shrink the tumor, thereby making liver resection not just more likely but also safer. They also enable assessing how well the patient responds to systemic treatment, which can inform the next steps in the therapeutic decision-making process [17].

Volumetric analysis and three-dimensional liver reconstruction are primarily aimed at evaluating the future liver remnant's viability and resectability and making surgical decisions [18]. These tools strive to precisely assess and analyze not only the liver's volume but also the vascular anatomy and the liver's functional reserve, thereby allowing a safe approach to be determined and enabling the integration of these tools into the planning of complex liver resections [19].

In contemporary treatment planning for sCRLMs, multidisciplinary tumor boards (MTBs) are vital and ensure a collective, patient-centered method for making treatment decisions [20]. When hepatobiliary surgeons, medical oncologists, radiation oncologists, radiologists, and interventional specialists assess disease extent, discuss treatment sequencing, and determine the optimal balance between surgical resection, systemic therapy, and local ablative treatments [21].

MTBs enhance patient selection for simultaneous vs. staged resections and conversion therapy in initially unresectable cases. They help us assess the feasibility of these interventions and assure us that systemic therapy is tailored to our patients based on tumor biology [7]. Multidisciplinary decision-making should be a cornerstone of managing sCRLM because it is crucial. That ensures that treatment plans are aligned with oncologic goals and an array of patient-specific factors [14].

Multidisciplinary decision-making is essential in sCRLMs management, as patient selection determines surgical feasibility and systemic therapy choices [20]. A team of surgeons, oncologists, radiologists, and interventional specialists in hepatobiliary medicine works together to evaluate the burden of the tumors, the condition of the liver and extrahepatic disease, and the patient's overall fitness to create treatment plans tailored to the individual [19].

For patients whose disease cannot be resected at first, conversion therapy can make it possible to remove the tumor later. This therapy includes chemotherapy and various targeted agents that work on different cancer pathways because they reduce tumor size [5]. However, they also enhance the cancer-cell vulnerability making resection more likely to succeed [9]. Integrating multidisciplinary expertise optimizes treatment strategies to maximize survival outcomes, minimize recurrence, and ensure the safest, most effective approach for each patient [12].

Despite these advances, significant challenges remain in optimizing outcomes for patients with sCRLM. These include identifying predictive biomarkers for treatment response, developing strategies to overcome chemotherapy resistance, and determining optimal treatment sequences for various patient subgroups. Additionally, the impact of various treatment approaches on quality of life and long-term survivorship represents an important area requiring further investigation. This systematic review aims to comprehensively evaluate recent developments in surgical techniques, systemic therapies, and local treatment modalities for sCRLM. By analyzing current evidence and emerging trends, we seek to provide evidence-based insights to guide clinical decision-making in this rapidly evolving field. Furthermore, we aim to identify knowledge gaps and areas requiring additional research to continue advancing the management of this challenging disease.

## Review

Aims and objectives

The primary aim of this systematic review is to evaluate and synthesize current evidence on the management of sCRLMs, focusing on surgical approaches, systemic therapy integration, and local treatment modalities. The specific objectives are to (1) compare outcomes between simultaneous and staged surgical approaches; (2) assess the role of minimally invasive techniques in sCRLM management; (3) evaluate the impact of various systemic therapy protocols on treatment outcomes; and (4) analyze the effectiveness of local treatment modalities in disease control.

Research question and protocol

The primary research question was developed using the PICO framework: “In patients with synchronous colorectal liver metastases, what are the comparative outcomes of different surgical approaches, systemic therapies, and local treatment modalities in terms of survival, morbidity, and quality of life?” (Table 1).

**Table 1:** PICO table P – population, I – intervention, C – comparison, O – outcome (e.g., pain, fatigue, nausea, infections, death)

Component	Description
Population	Adult patients (≥18 years) with synchronous colorectal liver metastases
Intervention	Surgical approaches (simultaneous/staged resection), systemic therapy, local treatments
Comparison	Between different surgical approaches, timing of interventions, types of systemic therapy, and local treatment modalities
Outcomes	Overall survival, disease-free survival, perioperative morbidity, quality of life, cost-effectiveness

Methodology

This systematic review was conducted in accordance with the Preferred Reporting Items for Systematic Reviews and Meta-Analyses (PRISMA) guidelines.

Eligibility Criteria

The review included studies published between January 2013 and December 2024 that focused on adult patients (≥18 years) with sCRLMs. Only English-language publications with full-text articles available were considered. We included randomized controlled trials, prospective and retrospective cohort studies, and case-control studies that reported at least one of the following outcomes: overall survival, disease-free survival, perioperative outcomes, or quality of life, with a minimum follow-up period of 12 months. Studies that focused on primary liver cancer (e.g., hepatocellular carcinoma) or non-CRLM hepatic malignancies were excluded. Case reports and series with fewer than 10 patients were excluded, along with studies focusing exclusively on metachronous liver metastases. We also excluded conference abstracts, letters to editors, review articles without original data, studies without clear methodology or outcome reporting, duplicate publications or overlapping patient populations, and animal studies or in-vitro research.

Search Strategy

A comprehensive literature search was conducted in multiple electronic databases, including PubMed/MEDLINE, EMBASE, Cochrane Library, and Web of Science, up to December 2024. The search strategy was designed to maximize sensitivity and included both MeSH terms and free-text keywords. Population-related terms included “colorectal cancer,” “colorectal neoplasm,” “liver metastasis,” “hepatic metastasis,” and “synchronous.” Intervention-related terms comprised “liver resection,” “hepatectomy,” “simultaneous resection,” “staged resection,” “chemotherapy,” “targeted therapy,” and “immunotherapy.” Outcome-related terms included “survival,” “morbidity,” “mortality,” “complications,” and “quality of life.” Boolean operators (AND, OR) were applied to refine search results. Additional sources included manual screening of reference lists, grey literature searches, and expert consultation. Study selection followed PRISMA guidelines, with two independent reviewers screening titles, abstracts, and full texts. Risk of bias was assessed using the Newcastle-Ottawa Scale for observational studies and the Cochrane Risk of Bias tool for randomized controlled trials, ensuring methodological rigor and reproducibility.

Study Selection

**Figure 1:**
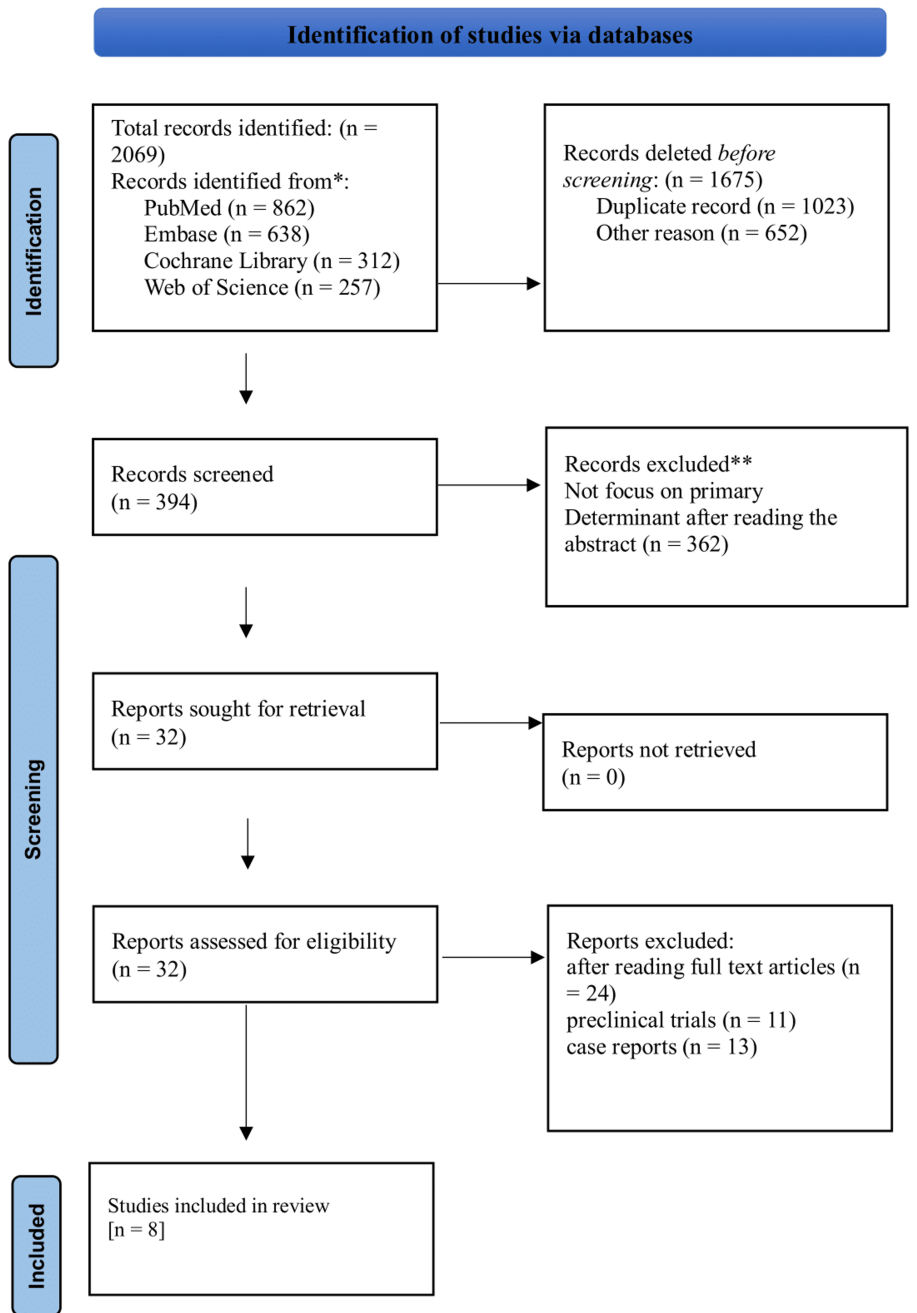
PRISMA chart PRISMA: Preferred Reporting Items for Systematic Reviews and Meta-Analyses

The study selection process involved two independent reviewers following a two-stage process: first the initial screening of titles and abstracts for potential eligibility and full-text review of potentially eligible studies. Any disagreements were resolved through discussion with a third reviewer. The selection process was documented using a PRISMA flow diagram (Figure 1), detailing the number of studies identified, screened, eligible, and included in the final analysis.

Outcome Measures

Primary outcomes included the overall survival, disease-free survival, and perioperative morbidity and mortality. Secondary outcomes include quality-of-life measures, cost-effectiveness, treatment-specific outcomes (response rates, conversion to resection), long-term oncologic outcomes, and technical feasibility measures.

Data Extraction and Quality of Studies

Data extraction was performed independently by two reviewers using a standardized form capturing study characteristics (design, period, location), patient demographics, disease characteristics, intervention details, outcome measures, and follow-up information. Quality assessment was conducted using the Mixed Method Appraisal Tool (MMAT) [22]. The quality assessment considered selection bias, performance bias, detection bias, attrition bias, reporting bias, and other potential sources of bias. Disagreements in quality assessment were resolved through discussion.

Results

The risk of bias is given in Table 2, where MMAT Score 4 indicates a medium risk of bias, MMAT Score 5 indicates a low risk of bias. The low risk shows that no significant issues related to bias in the study. High risk shows potential biases that may impact the study's internal validity. While unclear score shows the lack of sufficient information to assess the risk of bias in that domain.

**Table 2:** Risk of bias

Study ID	MMAT score	Selection bias	Performance bias	Detection bias	Attrition bias	Reporting bias	Other bias	Overall risk of bias
Kazi et al. (2023) [23]	4	Low	Low	High	Low	Unclear	None	Moderate
Chen et al. (2023) [24]	4	Low	Low	High	Low	Unclear	None	Moderate
Jo et al. (2024) [25]	4	Low	Low	High	Low	Unclear	None	Moderate
Dong et al. (2024) [26]	5	Low	Low	Low	Low	Low	None	Low
Mayo et al. (2013) [27]	5	Low	Low	Low	Low	Low	None	Low
Aghayan et al. (2023) [28]	5	Low	Low	Low	Low	Low	None	Low
Nadkarni et al. (2023) [29]	5	Low	Low	Low	Low	Low	None	Low
Endo et al. (2023) [30]	4	Low	Low	High	Low	Unclear	None	Moderate

Table 3 is organized and includes key information about study design, objectives, and main outcomes. The quality of the included studies was good to excellent as given in the MMAT column in Table 2.

**Table 3:** Characteristics of the included studies MMAT: Mixed Method Appraisal Tool

Sr. No.	Author & year	MMAT	Study type & method	Objective	Key outcomes
1	Kazi et al. (2023) [23]	4	Retrospective single-center study	To assess feasibility of simultaneous resection of synchronous CRLM	• 21.7% major complications rate • Safe for most patients • Caution needed for major hepatectomy with comorbidities
2	Chen et al. (2023) [24]	4	Secondary analysis of randomized clinical trial	To investigate association between liver metastases and immune checkpoint inhibitor efficacy	• Presence of liver metastases associated with worse outcomes • Better progression-free survival in patients without liver metastases • Higher disease control rate in non-liver metastasis group
3	Jo et al. (2024) [25]	4	Retrospective observational cohort study of 497 patients	To assess impact of primary tumor sidedness on prognosis in resectable CRLM	• No difference in prognosis based on sidedness • Key prognostic factors: CEA level, synchronous tumor, maximum tumor size
4	Dong et al. (2024) [26]	5	Multicenter prospective trial (ASSO-LM1)	To investigate resectability rate after XELOX and bevacizumab therapy	• 97% resectability rate • 66% objective response rate •Better survival with completed adjuvant treatment
6	Mayo et al. (2013) [27]	5	Multi-institutional database analysis	To investigate surgical management outcomes of synchronous CRLM	• Comparable morbidity and mortality between approaches • Similar long-term survival between simultaneous and staged • 20% overall complication rate
7	Aghayan et al. (2023) [28]	5	Single-center retrospective analysis	To evaluate laparoscopic parenchyma-sparing resection for large CRLM	• Similar operation time and conversion rates • Higher blood loss in large tumor group • Satisfactory short and long-term outcomes
8	Nadkarni et al. (2023) [29]	5	Technical report/case study	To present robotic left hepatectomy technique for CRLM	• Feasible procedure with 600cc blood loss • 340-minute operative duration • Successful discharge by day 7
9	Endo et al. (2023) [30]	4	Multi-institutional database analysis using win ratio approach	To compare simultaneous vs staged surgical treatment for synchronous CRLM	• Staged resection superior to simultaneous approach • Higher benefit in major hepatectomy cases • Win ratio of 1.59 favoring staged approach

Key Themes and Synthesis of Evidence

Surgical approach selection: Recent evidence from Endo et al. using the novel “win ratio” (WR) approach demonstrates the superiority of staged resection over the simultaneous approach (WR = 1.59). The advantage is particularly pronounced for major hepatectomies (WR = 1.93). However, Mayo et al.'s earlier multi-institutional study showed comparable morbidity, mortality, and long-term oncologic outcomes between approaches. Kazi et al. found simultaneous resection safe for selected patients, with major complications in 21.7% of cases. Key considerations include patient comorbidities significantly impact outcomes, especially in simultaneous major hepatectomies [23,27,30].

Evolution toward less invasive techniques is supported by recent evidence. Aghayan et al. demonstrated feasibility of laparoscopic parenchyma-sparing resections even for large (≥50mm) metastases. Nadkarni et al. showed successful implementation of robotic approaches with acceptable outcomes. Technical advantages include better visualization, reduced blood loss, comparable oncologic outcomes and faster recovery [28,29].

Systemic therapy integration: Chen et al. revealed important findings regarding immune checkpoint inhibitors: as the presence of liver metastases associated with worse outcomes, better progression-free survival in patients without liver metastases, and implications for patient selection and treatment sequencing [24].

ASSO-LM1 trial showed high resectability rate (97%) with XELOX + bevacizumab, significant objective response rate (66%) and improved survival with completed adjuvant treatment. The timing considerations for surgery post-bevacizumab are important (five-week interval recommended) [26].

Prognostic factors and patient selection: Jo et al. identified key prognostic factors includes CEA level, synchronous presentation and maximum tumor size. They suggest that primary tumor sidedness does not significantly impact prognosis [25].

Evolution of treatment paradigms: The trend toward a personalized approach based on patient factors (comorbidities, performance status), disease characteristics (tumor size, number, location), and technical considerations (resection extent, surgical approach). They suggest that it has a growing role in minimally invasive techniques and stressed the importance of multimodal therapy (systemic + local treatments).

Concluded Synthesis of Current Evidence

The reviewed studies demonstrate significant advancement in sCRLM management, with evolution toward more personalized approaches. Key findings suggest surgical approach should be individualized based on patient and disease factors. Minimally invasive techniques are increasingly viable even for complex cases. Systemic therapy plays a crucial role in optimizing outcomes. Patient selection and prognostic factor consideration are critical. Margin status and technical considerations remain important. Treatment sequencing requires careful consideration. These findings support a comprehensive, multimodal approach to sCRLM management, with emphasis on patient-specific factors in treatment selection.

Discussion

The management of sCRLMs has evolved significantly over the past decade through advances in multidisciplinary approaches. This systematic review synthesizes current evidence across four key domains: surgical approach timing, minimally invasive techniques, systemic therapy integration, and novel local treatments, while identifying prognostic factors that influence clinical decision-making.

Optimal Timing of Surgical Intervention: Simultaneous vs. Staged Resection

The debate regarding the optimal timing of primary and metastatic disease resection remains nuanced. Evidence supports both approaches in selected patients. Our analysis revealed that patient selection is the critical determinant of approach rather than the universal superiority of either strategy.

Recent high-quality evidence from Endo et al. supported staged resection through their novel WR analysis of 642 patients with sCRLM. Their study demonstrated a WR of 1.59 (95% CI 1.47-1.71), indicating that, in the hierarchical comparison of outcomes, the staged resection strategy was favored 1.59 times more often than the alternative approach. The confidence interval (CI) suggests a high degree of precision, as it does not include 1.0, reinforcing the statistical significance of the findings. This result supports the superiority of staged resection in improving key clinical outcomes for patients with sCRLM [29]. This suggests that the extent of liver resection significantly influences the safety profile of combined procedures.

However, these findings must be contextualized against the multi-institutional data from Mayo et al., who analyzed 1,004 patients and found comparable morbidity, mortality, and long-term oncologic outcomes between simultaneous and staged approaches [27]. The discrepancy between these studies likely stems from differences in patient selection criteria, institutional expertise, and supportive care protocols.

Machairas et al. provided valuable insights by identifying specific patient and disease factors that inform this decision [31]. Their analysis demonstrated that simultaneous resection may be preferable in patients with limited comorbidities, smaller hepatic tumor burden, and when both colorectal and liver procedures are technically straightforward. Conversely, staged approaches showed advantages in patients requiring complex hepatectomies or with significant medical comorbidities. This patient-tailored approach represents the current standard of care rather than a one-size-fits-all strategy.

The safety profile of simultaneous resections has been further validated by Kazi et al., who reported major complication rates of 21.7% (95% CI: 13.8%-31.5%) in a prospective series of 92 patients. Importantly, this study included a risk-stratification model that helps identify appropriate candidates for simultaneous resection [23].

Minimally Invasive Approaches for CRLM

The evolution of minimally invasive surgery has significantly impacted the management of sCRLM, with evidence now supporting both laparoscopic and robotic approaches in selected patients. The key question has shifted from whether these approaches are feasible to identifying which patients benefit most from these techniques.

The randomized trial by Aghayan et al. [28] provided level 1 evidence showing reduced complications with laparoscopy (p<0.01) and shorter hospital stays compared to open techniques [31]. When interpreting these findings, it is important to note that this study primarily included patients with limited tumor burden and favorable anatomic locations, potentially limiting generalizability to all sCRLM cases.

Regarding robotic approaches, Sijberden et al. demonstrated that robotic liver surgery (RLS) facilitated slightly higher “textbook outcome in liver surgery” (TOLS) rates than laparoscopic liver surgery (LLS) [32]. However, this study had important limitations, including a lack of long-term oncologic outcomes specific to sCRLM patients. The technical advantages of robotic surgery, including enhanced visualization and instrument articulation, must be weighed against increased costs and limited availability.

Integration of Modern Systemic Therapy

The integration of systemic therapy with surgical approaches has fundamentally changed sCRLM management paradigms, with contemporary evidence guiding both conversion strategies for initially unresectable disease and perioperative approaches for resectable disease.

The ASSO-LM1 trial reported by Dong et al. achieved a 97% resectability rate with preoperative XELOX and bevacizumab [26]. Their objective response rate of 66% with manageable toxicity (17% grade 3/4 diarrhea) demonstrates the potential of targeted therapy in the perioperative setting. This builds upon the foundation established by earlier studies while incorporating contemporary agents.

The concept of converting initially unresectable disease to resectable status has evolved significantly since Adam et al.'s seminal 2004 report. Current conversion chemotherapy regimens demonstrate substantially improved response rates and resectability outcomes. The TRIBE2 study showed improved progression-free survival with 5-FU, leucovorin, oxaliplatin, and irinotecan (FOLFOXIRI) plus bevacizumab (p<0.001), while the VOLFI trial reported an impressive response rate with modified FOLFOXIRI (mFOLFOXIRI) plus panitumumab in rat sarcoma virus (RAS) wild-type patients [33].

Importantly, molecular profiling now guides systemic therapy selection. Chen et al. demonstrated that immunotherapy efficacy varies based on metastatic patterns, with improved progression-free survival with durvalumab plus tremelimumab specifically in patients without liver metastases (HR, 0.54 (90% CI, 0.35-0.96)) [24]. This underscores the importance of considering the unique tumor microenvironment of liver metastases when planning systemic therapy.

For patients with potentially resectable disease, the timing and duration of perioperative chemotherapy remain debated. While the European perioperative oxaliplatin and capecitabine (EPOC) trial established the role of perioperative FOLFOX, contemporary practice has evolved to include targeted agents based on molecular profiling, highlighting the need for individualized approaches.

Novel Local Therapies and Prognostic Factors

Local therapy approaches for sCRLM have expanded beyond traditional surgical resection, with ablative techniques and intra-arterial therapies showing promise in selected patients. The chemo saturation with locator-assisted chemotherapy for colorectal liver metastases (CLOCC) trial provided level 1 evidence demonstrating improved overall survival with radiofrequency ablation plus chemotherapy compared to chemotherapy alone (p=0.01) for unresectable CRLM [34]. This landmark study established ablation as a standard treatment option for patients with limited tumor burden who are not surgical candidates.

The evaluation of drug-eluting beads with irinotecan (DEBIRI) by Jones et al. revealed an important limitation: radiological response did not predict pathological tumor response, emphasizing the need for careful interpretation of imaging following intra-arterial therapies [35].

Prognostic stratification has been refined through recent large-scale analyses. Jo et al. identified synchronous presentation (OR, 5.01; p<0.001), CEA levels (OR, 1.46; p=0.016), and maximum tumor size (OR, 1.09; p=0.026) as significant predictors of overall survival in their analysis of 497 patients with resectable CRLM [23]. These findings help identify patients who might benefit from more aggressive multimodal approaches.

## Conclusions

The management of sCRLM continues to evolve, with evidence supporting increasingly personalized approaches. While technical and therapeutic options have expanded significantly, careful patient selection and treatment sequencing remain critical to optimal outcomes. Future research should focus on the prospective validation of existing findings and the development of more precise patient selection criteria. These findings support a comprehensive, multimodal approach to sCRLM management, with emphasis on patient-specific factors in treatment selection. The integration of multiple treatment modalities, guided by evidence-based protocols and patient-specific factors, represents the current standard of care in this complex field.
